# Interactions between U and V sex chromosomes during the life cycle of *Ectocarpus*

**DOI:** 10.1242/dev.202677

**Published:** 2024-04-12

**Authors:** Jeromine Vigneau, Claudia Martinho, Olivier Godfroy, Min Zheng, Fabian B. Haas, Michael Borg, Susana M. Coelho

**Affiliations:** ^1^ Max Planck Institute for Biology, 72076 Tübingen, Germany; ^2^ Roscoff Biological Station, CNRS-Sorbonne University, Place Georges Teissier, Roscoff 29680, France

**Keywords:** UV systems, Algae, Sex chromosomes, Sexual dimorphism

## Abstract

In many animals and flowering plants, sex determination occurs in the diploid phase of the life cycle with XX/XY or ZW/ZZ sex chromosomes. However, in early diverging plants and most macroalgae, sex is determined by female (U) or male (V) sex chromosomes in a haploid phase called the gametophyte. Once the U and V chromosomes unite at fertilization to produce a diploid sporophyte, sex determination no longer occurs, raising key questions about the fate of the U and V sex chromosomes in the sporophyte phase. Here, we investigate genetic and molecular interactions of the UV sex chromosomes in both the haploid and diploid phases of the brown alga *Ectocarpus*. We reveal extensive developmental regulation of sex chromosome genes across its life cycle and implicate the TALE-HD transcription factor OUROBOROS in suppressing sex determination in the diploid phase. Small RNAs may also play a role in the repression of a female sex-linked gene, and transition to the diploid sporophyte coincides with major reconfiguration of histone H3K79me2, suggesting a more intricate role for this histone mark in *Ectocarpus* development than previously appreciated.

## Teaser

**Summary:** Investigation of genetic and molecular interactions between U and V sex chromosomes implicate OUROBOROS and histone H3K79me2 in developmental regulation of sex chromosome genes in different stages of the *Ectocarpus* life cycle.

## INTRODUCTION

The sex of an organism may be determined epigenetically, through environmental cues for example, or genetically by means of a sex locus or sex chromosome. A key factor that influences the nature of genetic sex determination is the stage of the life cycle when this occurs. In animals and flowering plants with XX/XY or ZW/ZZ systems, sex is determined during the diploid phase of the life cycle. However, in many other eukaryotes, such as bryophytes and most brown, red and green algae, sex is determined during a haploid phase of the life cycle called the gametophyte generation (Coelho et al., 2018). The brown algae provide a classic example of a haploid-diploid life cycle with U/V sex determination (Coelho et al., 2007, 2018). Haploid sex determination in these organisms occurs at meiosis and the inheritance of a U or V sex chromosome dictates whether the gametophyte develops into a female or a male (Coelho and Umen, 2021; Coelho et al., 2018). Once fully developed, the gametophytes undergo gametogenesis to produce gametes via mitosis. Once released into seawater, male gametes are attracted by a pheromone released from the female gamete (Maier and Muller, 1986), which then fuse to reconstitute a diploid genome in the sporophyte generation. Diploid sporophytes thus carry both a U and V sex chromosome yet are phenotypically ‘asexual’ because they bear no sexual characteristics, but rather undergo meiosis to re-initiate the haploid phase and complete the life cycle.

The brown alga *Ectocarpus* has recently emerged as a powerful model in which to study the structure and evolution of UV sex chromosomes. The *Ectocarpus* U and V chromosomes are characterized by a distinct sex-determining region (SDR) of similar size and structure that each contains about 20 genes (Ahmed et al., 2014; Coelho et al., 2019). Nine and eight of these genes are sex specific and are only present on the SDR of the U or V chromosome, respectively. A further 12 gametolog genes are shared by both SDRs, which are remnants of the ancestral pair of autosomes that gave rise to the UV sex chromosomes (Ahmed et al., 2014; Lipinska et al., 2017). Although recombination is suppressed within the SDRs, the U and V recombine along pseudoautosomal regions (PARs) that flank the centrally located SDR (Avia et al., 2018; Luthringer et al., 2015b). Compared with autosomes, the PAR tends to harbour an excess of evolutionarily young or taxonomically restricted genes whereas each SDR is highly enriched for repeats and transposable elements (TEs) (Ahmed et al., 2014; Luthringer et al., 2015b). The unique genetic makeup of the PAR and SDR is also reflected by a highly distinct chromatin landscape compared with autosomes, being enriched for repressive histone H3K79 and H4K20 methylation in *Ectocarpus* (Gueno et al., 2022).

Theoretical models have long predicted that genes within the SDR would have sex-specific functions and would be expressed specially in the haploid phase of the life cycle (Bull, 1978). Indeed, expression profiling in UV systems such as brown algae and moss has revealed preferential expression of SDR genes in the gametophyte stage (Ahmed et al., 2014; Avia et al., 2018; Carey et al., 2021; Lipinska et al., 2017). Given that phenotypic sex is not expressed in the sporophyte generation, it is unlikely that SDR genes would be required during the diploid phase of the life cycle, although such predictions have yet to be tested experimentally. Indeed, very few studies have addressed the potential genetic interactions between U and V sex-linked genes.

Brown algae are an ideal system in which to study interactions between the U and V chromosomes because ploidy levels are not always correlated with a particular stage in the life cycle. For example, the gametes in several algal species can develop into haploid sporophytes through parthenogenesis (Arun et al., 2019; Bothwell et al., 2010). Conversely, the gametophyte generation is continuously repeated regardless of ploidy in mutants of the *OUROBOROS* (*ORO*) gene, which encodes a TALE-HD transcription factor that is required for the gametophyte-to-sporophyte transition in *Ectocarpus* (Arun et al., 2019; Coelho et al., 2011). Strikingly, diploid *oro;oro* mutants bear both a U and V sex chromosome, yet phenotypically behave like a male gametophyte, suggesting that the SDR of the male V chromosome is genetically dominant over that of the female U (Ahmed et al., 2014). This is suggestive of a master regulatory gene on the male SDR that triggers male development and/or suppresses female development, which has recently been shown to be a male SDR-specific gene encoding a high-mobility group (HMG) transcription factor (Lipinska et al., 2017; Luthringer et al., 2024; Müller et al., 2021). The observation that the V acts dominantly over the U in a gametophyte context thus provides a key opportunity to gain further insight into the genetic and molecular mechanisms underlying UV sex determination. How are SDR genes and sexual differentiation programmes regulated across the life cycle? What is the molecular basis for the male gametophyte-like phenotype of *oro;oro* mutants and what can this tell us about the genetic interactions of the U and V chromosomes? And given its pervasive role in XX/XY sex determination systems, does any form of dosage compensation also occur on the U and V sex chromosomes during the diploid sporophyte generation?

Here, we examine the developmental regulation and intricate relationship of the U and V sex chromosomes across the life cycle of *Ectocarpus.* To unravel these genetic interactions, we investigate gene expression and chromatin state in both wild type (WT) and *oro* mutants. We show how sex chromosome genes are developmentally regulated across the life cycle, with female sex-linked genes strongly repressed in the diploid phase. We further identify at least one male sex-linked gene that shows dosage compensation in the sporophyte. Despite the homozygous loss of ORO function in the diploid phase resulting in a male gametophyte-like phenotype, these mutants exhibit feminized transcriptional patterns, suggesting that ORO activity is required to suppress the expression of sex-linked genes on both the U and V sex chromosome. SDR gene regulation in the sporophyte does not appear to involve local changes in chromatin state but may involve small RNA silencing at one female-specific gene. Finally, we show how histone H3K79me2 modifications are re-configured across the *Ectocarpus* life cycle, with drastic reductions observed over the SDR of the U and V sex chromosomes. Contrary to its pattern in the gametophyte generation, H3K79me2 deposition does not correlate with repressed genes in the sporophyte, suggesting a distinct role for this histone mark in the diploid phase of the life cycle.

## RESULTS

### ORO is required to suppress male sex determination in the diploid phase of the life cycle

**Fig. 1.**
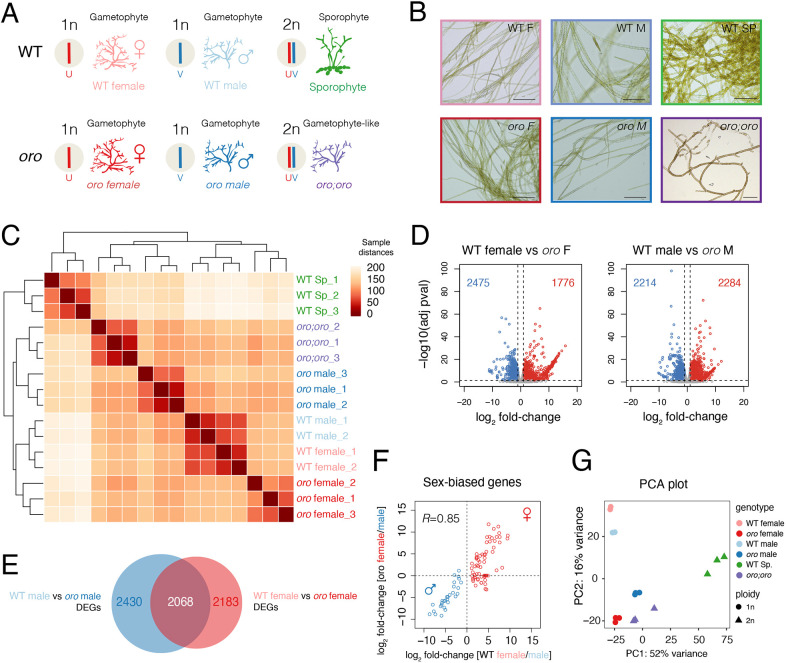
**Transcriptional profiling across the *Ectocarpus* life cycle.** (A) Schematic summarizing the genotypes profiled in this study. (B) Representative images of each genotype illustrating the gametophyte-like phenotype of diploid *oro;oro* mutants. Images are representative of 20 samples. Scale bars: 100 µm. (C) Sample distance matrix of the RNA-seq datasets generated and analysed in this study. (D) Volcano plots summarizing DEGs between WT and *oro* female (left) and male (right) gametophytes. Upregulated (log_2_ fold-change>1 and adjusted *P*-value<0.05) and downregulated (log_2_ fold-change<−1 and adjusted *P*-value<0.05) genes are highlighted in red and blue, respectively. The total number of DEGs are indicated in each plot. (E) Venn diagram summarizing the overlap of DEGs between WT and *oro* female and male gametophytes. (F) Plot of the pairwise correlation of the differential expression (log_2_ fold-change) of SBGs between females and males in a WT and *oro* background. Female and male SBGs are highlighted in red and blue, respectively. Pearson's correlation coefficient is shown. (G) Principal component analysis illustrating the variation among the RNA-seq datasets from WT females (*n*=2 replicates) (Gueno et al., 2022), WT males (*n*=2 replicates) (Gueno et al., 2022), WT sporophytes (Sp.) (*n*=3 replicates), haploid *oro* females (*n*=3 replicates), haploid *oro* males (*n*=3 replicates) and diploid *oro;oro* mutants (*n*=3 replicates).

To investigate interactions between the U and V sex chromosomes, we exploited *oro* mutant lines in *Ectocarpus* (Fig. 1A) (Coelho et al., 2011). We have shown previously how haploid male and female *oro* gametophytes largely phenocopy WT gametophytes at a morphological and functional level (Fig. 1B) (Arun et al., 2019; Coelho et al., 2011). To explore how the loss of *oro* function impacts these phenotypic differences at the molecular level, we compared gene expression of *oro* mutants with WT gametophyte lines by RNA sequencing (RNA-seq) (Fig. 1C,D; Table S1). We observed a large number of differentially expressed genes (DEGs) in *oro* males and *oro* females compared with WT gametophytes of the equivalent sex (Fig. 1D; Table S1). Around a half of these ORO-dependent DEGs were common to both sexes (46.0% in males; 48.6% in female) (Fig. 1E), suggesting that many of the transcriptional changes observed in the absence of a functional ORO protein largely occur independently of sexual differentiation. Consistent with this, the sex bias of effector genes involved in specifying a WT sexual phenotype (i.e. sex-biased genes) remained sex-biased in *oro* mutants (Fig. 1F), indicating that *oro* gametophytes retain male and female characteristics at the transcriptional level. Taken together, these results confirmed that the *oro* mutation does not affect the sexual identity of haploid gametophytes.

Male and female *oro* gametophytes were crossed to obtain a diploid (UV) *oro;oro* mutant line (Ec581) (Ahmed et al., 2014). This allowed us to combine the U and V chromosomes within the same gamete-producing individuals (Fig. 1A). Consistent with continuous cycling of gametophytes in *oro* lines via parthenogenesis (Coelho et al., 2011), diploid *oro;oro* mutants also retained gametophyte-like characteristics, including richly branched, wavy thalli and a lack of substrate adherence (Fig. 1B). This was also reflected at the level of gene expression, with diploid *oro;oro* mutants more similar to haploid gametophytes than to WT diploid sporophytes (Fig. 1C,G). Test crosses with female lines suggested that the diploid *oro;oro* line behaves as a functional male gametophyte (Fig. S1), consistent with previous results (Ahmed et al., 2014). Thus, the homozygous loss of ORO in diploid lines results in functional gametophytes that phenocopy male sex characteristics, despite the presence of both the U and V sex chromosome.

### Developmental transcription factors undergo dynamic regulation across the life cycle

**Fig. 2.**
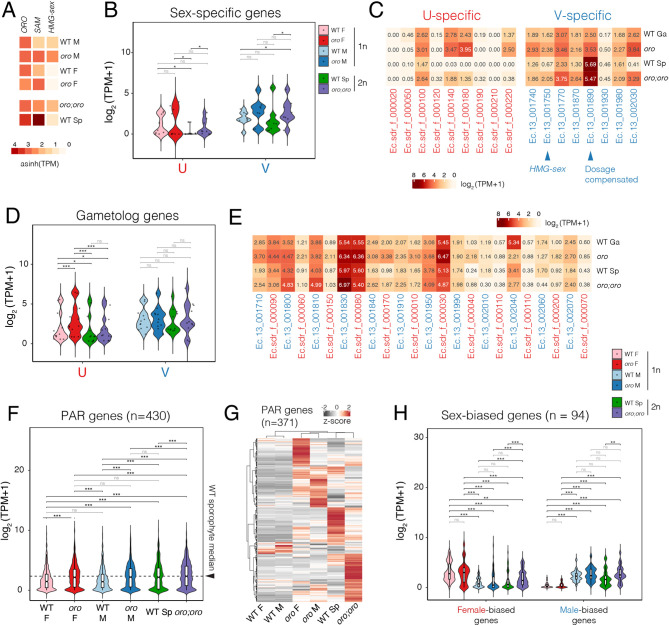
**Transcriptional dynamics of the sex chromosomes across the *Ectocarpus* life cycle.** (A) Expression of the transcription factors genes *ORO*, *SAM* and *HMG-sex* across the WT and *oro* genotypes and life stages. Expression represents the inverse hyperbolic sine (asinh) transform of the mean RNA-seq TPM values. The mean value of the biological replicates is shown. WT females (*n*=2 replicates) (Gueno et al., 2022); WT males (*n*=2 replicates) (Gueno et al., 2022); WT sporophytes (Sp) (*n*=3 replicates); haploid *oro* females (*n*=3 replicates); haploid *oro* males (*n*=3 replicates); and diploid *oro;oro* mutants (*n*=3 replicates). (B-E) Violin plots and heatmaps summarizing the expression of genes specific to the sex-determining region (SDR) of the female U and male V sex chromosomes (B,C) and the expression of gametolog genes (D,E). Expression represents the log_2_ of the mean RNA-seq TPM+1 values. (F,G) Violin plot and heatmap summarizing the expression of genes located on the pseudoautosomal region (PAR). Expression in the violin plot represents the log_2_ of the mean RNA-seq TPM+1 values, and the heatmap represents z-score normalized RNA-seq TPM values. (H) Violin plot summarizing the expression of sex-biased genes (*n*=94). Expression represents the log_2_ of the mean RNA-seq TPM+1 values. Statistical pairwise comparisons indicated on the violin plots represent the *P*-value of paired Student's *t*-tests (B,D,F) or paired Wilcoxon signed-rank tests (H). *P<0.05, **P<0.01, ***P<0.001. ns, no significance.

The phenotype observed in diploid *oro;oro* gametophyte-like mutants indicates that the V chromosome is genetically dominant and leads to a male sexual phenotype. This suggests that the V chromosome harbours a dominant genetic factor that is responsible for specification of male gametophytic fate. Consistent with this idea, a gene present on the V chromosome encodes an HMG transcription factor called HMG-sex that acts a master regulator of male sex determination in *Ectocarpus* and kelps (Luthringer et al., 2024). Consistent with this, *HMG-sex* transcripts were barely detectable in WT sporophytes compared with male gametophytes (Fig. 2A-C). Interestingly, whereas *HMG-sex* expression was reduced in the WT sporophyte, it was increased twofold in both haploid and diploid *oro* mutants compared with the WT male gametophyte (Fig. 2A). This suggests that ORO activity may be required to suppress *HMG-sex* expression in the WT sporophyte in a manner similar to other V-linked genes (Fig. 2B,C) (Luthringer et al., 2024). Given the role *HMG-sex* plays in male sex determination (Luthringer et al., 2024), its increased expression in diploid *oro;oro* mutants likely explains the male sex phenotype observed in these lines. Indeed, an *hmg-sex* knockout in diploid *oro;oro* mutants converts male sex to a female phenotype (Luthringer et al., 2024), providing further indication for a link between ORO activity and the repression of *HMG-sex* in the diploid sporophyte.

Whereas *ORO* expression is fairly stable across major stages of the *Ectocarpus* life cycle, *SAMSARA* (*SAM*) has a complementary expression pattern compared with *HMG-sex* (Fig. 2A). *SAM* encodes a TALE-HD transcription factor that is also required for the gametophyte-to-sporophyte transition in *Ectocarpus* (Arun et al., 2019). The similar phenotype of *oro* and *sam* mutants, coupled with the fact that ORO and SAM can interact *in vitro* (Arun et al., 2019)*,* suggests that they form a heterodimer to exert their function, similarly to other TALE-HD transcription factors (Dierschke et al., 2021). Both *ORO* and *SAM* had the highest expression level in the WT sporophyte generation, which also happens to coincide with downregulation of *HMG-sex* (Fig. 2A), suggesting that ORO/SAM activity is associated with repression of *HMG-sex* expression, be this directly or indirectly.

### UV sex chromosome genes are developmentally regulated across the *Ectocarpus* life cycle

Genes that function to specify a female or male gametophyte are predicted to be retained within the SDR of the U or V sex chromosomes, respectively. In contrast, sporophyte-specific genes required in the diploid phase would be lost from one of the SDRs if hemizygosity were to not greatly reduce fitness (Bull, 1978). To evaluate these predictions and further understand the mechanism underlying interactions between the U and V chromosomes, we examined the patterns of sex chromosome gene expression across several stages of the *Ectocarpus* life cycle.

In WT sporophytes, in which no sex is determined, transcript abundance of both U- and V-specific genes (i.e. genes that are present specifically on the female or male SDR, respectively) was reduced compared with WT gametophytes, with U-specific genes being largely silenced (Fig. 2B,C). Only one of the nine U-specific genes remained appreciably expressed [transcripts per million (TPM)>1] in WT sporophytes, compared with five of the eight V-specific genes (Fig. 2C). In contrast, V-specific genes were significantly upregulated in haploid *oro* mutants compared with WT male gametophytes (Fig. 2B,C). Interestingly, the expression of both U- and V-specific genes was significantly increased in diploid *oro;oro* mutants compared with WT sporophytes, with V-specific genes regaining transcript levels similar to those in WT male gametophytes (Fig. 2B,C). ORO activity thus appears to be required to suppress SDR gene expression during the diploid phase of the life cycle, further explaining the gametophyte-like identity of *oro;oro* mutants.

In organisms with XX/XY and XO sex determination, chromosome-scale dosage compensation of the X chromosome occurs to equalize the expression of X-linked genes (Disteche, 2012; Meller, 2000). We thus wondered whether a similar phenomenon occurs in a UV sex determination system. Although chromosome-scale dosage compensation of the U and V would not be required during the haploid phase of the life cycle, dosage compensation could take place at the single gene level for U- or V-linked genes that are required in the diploid sporophyte generation. Closer examination of the genes located on the SDR of each sex chromosome revealed one such gene on the male SDR encoding a hypothetical protein with a transmembrane domain (*Ec-13_001890*; Fig. 2C), which had twofold increased expression in diploid sporophytes compared with the haploid (male) stage. No dosage compensated genes were identified among U-specific genes. Thus, dosage compensation of SDR genes appears to be rare in the diploid phase of the *Ectocarpus* life cycle.

We next examined the expression of gametolog genes, which represent a class of homologous gene pairs present on the SDR of both the U and V chromosomes (Ahmed et al., 2014). Interestingly, gametologs on the U chromosome were significantly downregulated in WT sporophytes compared with WT female gametophytes (Fig. 2D,E). In contrast, no significant changes in transcript abundance were observed across the life cycle for V gametologs (Fig. 2D,E). Moreover, unlike U-specific genes, U gametologs were not significantly upregulated in diploid *oro;oro* mutants compared with WT sporophytes (Fig. 2D,E). This observation suggests that the presence of both sex chromosomes in the sporophyte is associated with the silencing of some U (but not V) gametologs. Together, our results indicate that genes located within the SDR of both the U and V chromosome are predominantly expressed in the gametophyte generation, with those on the U chromosome most strongly repressed in the diploid phase.

We extended our analysis to genes present within the PARs of the sex chromosomes, which are identical in both the U and V chromosomes (Avia et al., 2018; Luthringer et al., 2015b). Consistent with our previous findings (Luthringer et al., 2015b), PAR genes had higher levels of expression in WT sporophytes compared with WT male or female gametophytes (Fig. 2F). Interestingly, PAR transcripts were significantly upregulated in *oro* male or female gametophytes compared with their WT equivalent (Fig. 2F), suggesting that ORO may be required to suppress PAR gene expression in the haploid phase of the life cycle. PAR gene expression was also modestly but significantly increased in diploid *oro;oro* mutants compared with WT sporophytes (Fig. 2F), indicating that ORO may also modulate PAR gene expression in the diploid phase of the life cycle albeit to a lesser degree than in the haploid phase. Indeed, closer examination of PAR gene expression revealed that the majority (86.2%; 371 of 430) are subjected to substantial ORO-dependent repression in both haploid and diploid phases (Fig. 2G). Thus, unlike the attenuated expression of SDR genes in the diploid phase, PAR genes appear to be downregulated during the haploid phase of the life cycle in an ORO-dependent manner.

### Autosomal sex-biased genes are repressed in the sporophyte generation

We further assessed the impact of increased ploidy on the expression of sex-biased genes (SBGs), which are defined as autosomal genes that are differentially regulated between male and female gametophytes. We considered a conservative set of 94 SBGs that were sex-biased in at least two out of three independent datasets (Table S2) (Gueno et al., 2022; Lipinska et al., 2015). SBGs are unlikely to be required in the sporophyte generation given the lack of expression of sexual phenotypes during this stage of the life cycle (Luthringer et al., 2015a). Indeed, both male and female SBGs were significantly downregulated in WT sporophytes compared with gametophytes of the equivalent sex (Fig. 2H). As was observed for genes specific to the SDR of the female U chromosome (Fig. 2B), the strong reduction in transcript levels in WT sporophytes was more pronounced for female SBGs compared with male SBGs (Fig. 2H). Interestingly, male SBG expression was once more increased in diploid *oro;oro* mutants compared with WT sporophytes, reaching levels similar to those in the WT male gametophyte (Fig. 2H). Thus, the expression of V-specific genes and male SBGs in diploid *oro;oro* gametophytes resembles that in haploid male gametophytes, likely explaining the ability of *oro;oro* mutants to function as fertile male (Fig. S1).

Despite diploid *oro;oro* mutants functioning as male gametophytes, diploid *oro;oro* mutants also appear to be ‘feminized’ by the increased expression of a subset of female SBGs (Fig. 2H), which is likely reflective of the presence of the U chromosome in diploid gametophytes, indicating complex interplay between the differentiation programmes governing each sex. In contrast, no significant differences in SBG expression were observed between WT and *oro* haploid gametophytes (Fig. 2H). Thus, consistent with the presence of both a U and V sex chromosome, *oro;oro* gametophytes phenocopy male sex, but molecularly display a slightly feminized transcriptome (Fig. 2H). These results further emphasize how ORO activity is required to suppress gametophytic fate in the diploid phase of the life cycle via the repression of both sex-specific and sex-biased genes.

### SDR gene regulation is largely independent of sRNA silencing and local chromatin changes

**Fig. 3.**
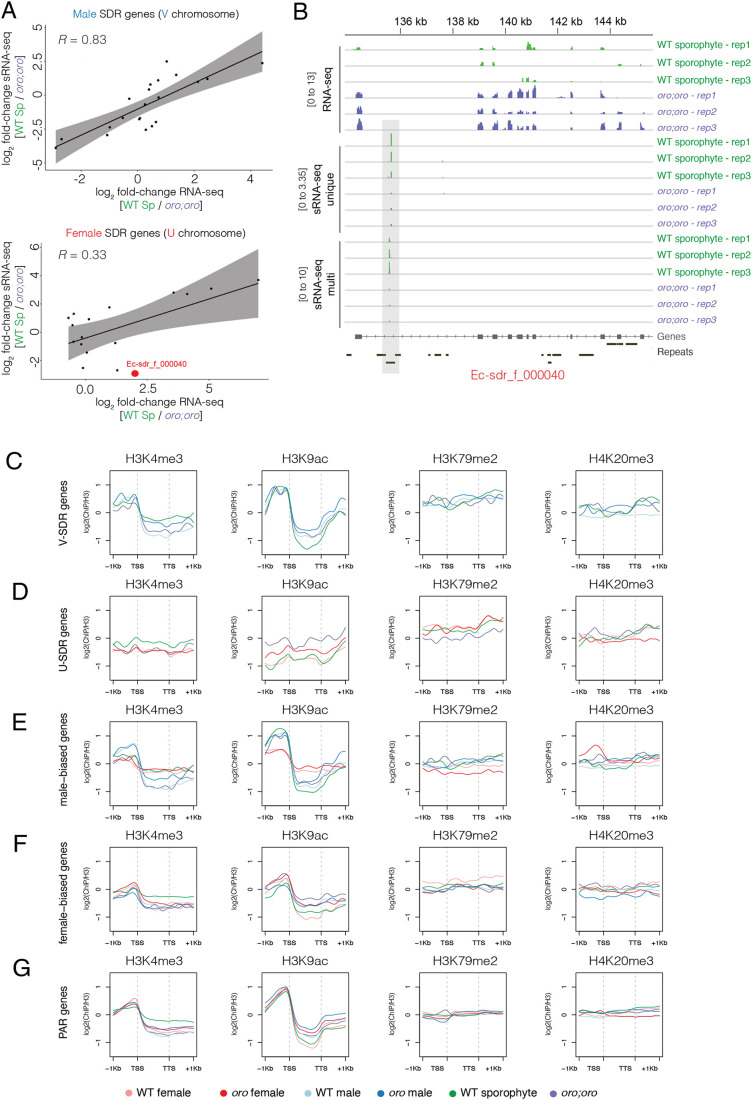
**The small RNA and chromatin state of genes involved in sex determination.** (A) Plot of the pairwise correlation of the differential expression (log_2_ fold-change) of male (top) and female (bottom) SDR genes and their associated small RNAs between diploid WT sporophytes and *oro;oro* mutants. Spearman's correlation coefficient is shown. Highlighted in red is the female-specific gene *Ec-sdr_f_000040*, which is significantly upregulated in *oro;oro* (log_2_ fold-change=1.98; *P=*0.005) and has a corresponding significant decrease in small RNA levels (log_2_ fold-change=−2.89; *P=*4.08×10^−11^) (Wilcoxon signed-rank tests). Sp, sporophyte. (B) A genome browser view of the *Ec-sdr_f_000040* locus showing RNA-seq coverage alongside unique and multi-mapped small RNAs in diploid WT sporophytes and *oro;oro* mutants. Dynamic small RNAs are highlighted with grey shading. (C-G) ChIP-seq signals of H3K4me3, H3K9ac, H3K79me2 and H4K20me3 over SDR genes of the male V sex chromosome (C), SDR genes of the female U sex chromosome (D), male-biased genes (E), female-biased genes (F), and PAR genes (G). ChIP-seq signals represent the log_2_ ratio of immunoprecipitated DNA relative to histone H3 and are colour-coded based on the key at the bottom to differentiate between the four genotypes profiled in the study and WT male and female gametophyte data published previously (Gueno et al., 2022).

Small RNAs (sRNAs) play an important role in the regulation of gene expression and TE silencing in eukaryotes (Chen, 2009; Rana, 2007). We therefore investigated whether the differential expression of SDR genes between WT sporophytes and *oro;oro* diploid mutants was associated with differences in sRNA accumulation (Table S3). Overall, sRNAs do not appear to explain gene expression changes at the female SDR (Fig. S2). Conversely, global sRNA accumulation of multi-mapped sRNA was moderately but significantly decreased in *oro;oro* compared with the WT sporophyte at the male SDR (Fig. S2B). Accordingly, one-third (8/23; 34.8%) of the sRNAs associated with genes on the male SDR were significantly downregulated (*P*<0.05 and log_2_ fold-change<−1.2) in *oro;oro* mutants compared with the WT sporophyte (Table S3). Although six of these eight genes encoded gametologs (i.e. genes with a homologue on the female SDR), there was no significant enrichment for gametologs among downregulated genes (Chi-square test *P*=0.3537; Table S3). To assess further whether sRNAs are negative regulators of SDR gene expression, we calculated the correlation coefficient of the fold-change difference in sRNA accumulation and mRNA abundance between WT sporophytes and *oro;oro* mutants (Fig. 3A). Surprisingly, sRNA accumulation was positively correlated with male SDR gene expression (Fig. 3A), suggesting that sRNAs may not be required for repression of male SDR genes in WT sporophytes (Fig. 3A). In contrast, one female SDR gene (*Ec-sdr_f_000040*) that was specifically upregulated in *oro;oro* happened to be associated with decreased accumulation of sRNAs (Fig. 3B). These sRNAs accumulated to a higher extent in WT sporophytes within the last intron of the locus and overlapped with an unclassified repeat (Fig. 3B). Thus, other than one U-specific gene within the female SDR, our observations indicate no clear association between the presence of sRNAs and the silencing of SDR genes.

Recent studies have shown how the re-configuration of histone modifications during sexual differentiation are intimately linked with the establishment and/or maintenance of sex-specific transcriptional programmes (Brown and Bachtrog, 2014; Gueno et al., 2022). We thus examined whether the differential deposition of histone modifications was also associated with transcriptional changes of sexual differentiation programmes among the different life cycle contexts. Our previous work in *Ectocarpus* has shown that H3K4me3 and H3K9ac are associated with the transcription start sites (TSSs) of active genes, whereas H3K79me2 and H4K20me3 deposition is correlated with decreased transcript abundance (Bourdareau et al., 2021; Gueno et al., 2022). We thus generated chromatin immunoprecipitation followed by sequencing (ChIP-seq) profiles for H3K4me3, H3K9ac, H3K79me2 and H4K20me3 in the different life cycle stages and compared these with our previously published profiles of WT gametophytes (Fig. S3). We validated the robustness of our ChIP-seq data in two ways. First, we confirmed high correlation between the replicates of each histone mark in each life cycle stage (Fig. S3A-F). Second, we confirmed that the deposition of active and repressive marks was positively and negatively correlated with gene expression, respectively (Fig. S3G-L).

Next, we aggregated ChIP-seq coverage for each histone mark over the different groups of genes involved in sex determination, i.e. over U- and V-specific genes and SBGs (Fig. 3C-G). Surprisingly, no major differences in histone mark deposition were evident that could explain the transcriptional changes we observed among the different life cycle contexts for genes located both within the male and female SDR and along the PAR (Figs 2D-G, 3C,D,G). However, the upstream promoter region of male SBGs clearly showed reduced levels of active H3K4me3 and H3K9ac marks in WT and *oro* female gametophytes compared with their male equivalent, consistent with the reduced expression of male SBGs in females (Fig. 2H; Fig. 3E). Similarly, the upstream promoter region of female SBGs also showed reduced levels of H3K4me3 and H3K9ac in WT male gametophytes and WT sporophytes (Fig. 3F), consistent with the reduced expression of female SBGs in these stages (Fig. 2H). Thus, although some sex-biased genes appear to undergo dynamic changes in the level of active histone marks between male and female gametophytes, consistent with our previous findings (Gueno et al., 2022), local *cis* changes at chromatin do not appear to coincide with changes in the expression of U and V sex chromosomes genes.

### The landscape of H3K79me2 is re-configured across the *Ectocarpus* life cycle

**Fig. 4.**
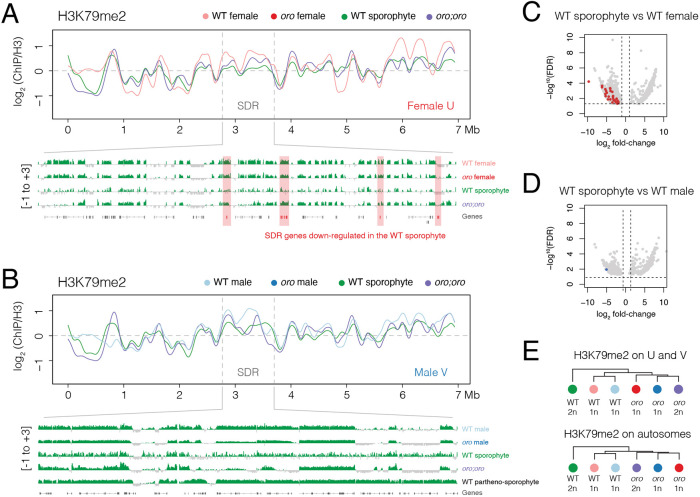
**H3K79me2 is re-configured across the *Ectocarpus* life cycle.** (A,B) Distribution of H3K79me2 over the female U (A) and male V (B) sex chromosome. The position of the sex-determining region (SDR) of each sex chromosome is indicated between the grey dashed lines. A genome browser view of H3K79me2 enrichment over the complete SDR is provided below. Signals represent the ChIP-seq log_2_ enrichment of immunoprecipitated H3K79me2-associated DNA relative to histone H3 calculated in 10 kb bins. (C,D) Volcano plots of differential enrichment of H3K79me2 peaks between the WT sporophyte and WT female gametophyte (C) and the WT sporophyte and WT male gametophyte (D). Peaks associated with female and male SDR genes are highlighted in red and blue, respectively. (E) Hierarchical clustering of H3K79me2 profiles over sex chromosomes and autosomes.

We next considered whether more global changes in chromatin could explain the silencing of SDR genes in the WT sporophyte generation. Our previous work showed that the repeat-rich sex chromosome in *Ectocarpus* is highly enriched for chromatin signatures defined by repression-associated H3K79me2 marks (Gueno et al., 2022). Consistent with this, we observed very broad domains of H3K79me2 enrichment along the U and V sex chromosomes, with the majority of the SDRs being covered by H3K79me2 in the WT female and male gametophyte (Fig. 4A,B). In the WT sporophyte, these broad domains of H3K79me2 were reduced in intensity compared with the gametophyte generations, with the female SDR showing a particularly pronounced reduction in H3K79me2 levels (Fig. 4A,B). Differential analysis of H3K79me2 peak enrichment confirmed this, with the majority (17/22; 77.3%) of U-specific genes associated with one or more peaks that were significantly depleted in H3K79me2 levels in the female gametophyte compared with the WT sporophyte (Fig. 4C; Table S4). Only one V-specific gene showed this association (Fig. 4D; Table S5), consistent with a more modest decrease in H3K79me2 along the male SDR (Fig. 4B).

In contrast, the strong reduction of H3K79me2 within the SDR was much less pronounced in diploid *oro;oro* mutants, which instead more strongly resembled that seen in WT male and female gametophytes (Fig. 4A,B). Similarly, this reduction was not evident in the SDR of a haploid male partheno-sporophyte (Fig. 4B) (Bourdareau et al., 2021), suggesting that reconfiguration of H3K79me2 only occurs in the diploid sporophyte, in the presence of the U chromosome. Hierarchical clustering of H3K79me2 profiles on the U and V sex chromosomes further illustrates how WT sporophytes are clearly distinct from the other life cycle stages, which was also evident along autosomes (Fig. 4E). Thus, the union of the U and V sex chromosome in the sporophyte generation results in a reduction of H3K79me2 along the SDRs, which is once again increased in the gametophyte-like context of diploid *oro;oro* mutants. The reconfiguration of H3K79me2 is further accompanied by the downregulation of SDR genes in the WT sporophyte generation (Fig. 2B-D), suggesting that this could impact the transcriptional state of the U and V sex chromosomes. Closer inspection of H3K79me2 deposition on genes grouped by expression level in the WT sporophyte illustrated a poorer correlation with repressed genes compared with other life stages (Fig. S3F), further suggesting that the repressive role of H3K79me2 might not extend to the diploid phase of the life cycle.

## DISCUSSION

Eukaryotic organisms that are characterized by an alternation of generations must undergo dynamics changes in genomic activity to specify distinct developmental programmes in the alternating haploid and diploid stages of their life cycle (Coelho et al., 2007). As a haploid gametophyte, an individual will grow vegetatively through haploid mitosis and eventually initiate gametogenesis to mate with the opposite sex. A diploid sporophyte will also grow vegetatively through diploid mitoses, but will instead initiate meiosis to begin the process of sporulation. Thus, only diploid sporophytes should activate the meiotic programme, and haploid gametophytes alone should transition towards gametogenesis. Tight control of these processes must thus be ensured to trigger the correct developmental programme at the opportune moment in the life history (Perrin, 2012). Here, we have examined the developmental regulation and intricate relationship of U and V sex chromosomes across the life cycle of the model brown alga *Ectocarpus*.

TALE HD transcription factors are involved in controlling the diploid transition across eukaryotic lineages (Arun et al., 2019; Dierschke et al., 2021; Kariyawasam et al., 2019), whereas sex chromosome genes are predicted to regulate haploid gametophyte development (Bachtrog, 2006; Charlesworth, 2016; Coelho and Umen, 2021). Consistent with this notion, genes involved in haploid-to-diploid transitions and sex determination, including those on the sex chromosomes, undergo extensive transcriptional reprogramming across the *Ectocarpus* life cycle. This includes major developmental regulators such as the TALE HD transcription factors ORO and SAM (Arun et al., 2019; Coelho et al., 2011) and the HMG-domain transcription factor HMG-sex.

In male gametophytes, female-biased gene (FBG) expression remained unchanged compared with the WT sporophyte, suggesting that the male programme appears to be driven solely by upregulation of MBGs. In contrast, female gametophytes appear to both strongly downregulate male-biased genes (MBGs) and strongly upregulate FBGs. Thus, SBGs undergo dynamic and opposing changes in expression during female sexual development, whereas an increase in MBG expression alone appears to be sufficient for male sexual development. This trend is consistent with the male sex determination programme acting dominantly over the female programme. Moreover, the persistent expression of some FBGs in males suggests that they might also be involved and/or required for aspects of male development. This potential pleiotropic effect of FBGs is further reflected by the fact that FBGs have a broader expression pattern than MBGs in *Ectocarpus* (Cossard et al., 2022; Lipinska et al., 2015). Stronger pleiotropic constraints on the broad expression of FBGs has been shown to be prevalent even among animal models such as *Drosophila* (Allen et al., 2018). Our results are consistent with female sex determination acting as the ‘background’ programme of sexual development in *Ectocarpus*, which is then suppressed through the expression of male-dominant regulatory factors (Ahmed et al., 2014; Avia et al., 2018; Müller, 1967; Müller et al., 2021).

Because the U and V sex chromosomes function during the haploid phase of the life cycle, chromosome-scale dosage compensation is not expected to occur as it would in XX/XY sex determination systems (Duan and Larschan, 2019). Nevertheless, we identified one V-specific gene encoding a hypothetical transmembrane protein that appears to be subjected to dosage compensation in the sporophyte generation, suggesting that it might also play an important role in both haploid and diploid phases of the life cycle.

The diploid homozygous loss of the TALE-HD transcription factor ORO results in a male gametophyte-like phenotype that produces functional male gametes capable of undergoing fertilization, despite the presence of both the U and V sex chromosome (Ahmed et al., 2014). This is consistent with the presence of the male sex-determining gene *HMG-sex* on the SDR of the V chromosome (Luthringer et al., 2024). Interestingly, we observed that *HMG-sex* expression is de-repressed in *oro;oro* mutants, suggesting that the regulatory activity of ORO is required to repress *HMG-sex* expression, be this directly or indirectly. Moreover, diploid *oro;oro* mutants do not fully silence the female developmental programme as these lines are transcriptionally ‘feminized’ in comparison with a WT male gametophyte. This is presumably also due to the inability to silence SDR genes in the absence of ORO activity, which would normally occur in the diploid sporophyte generation. Our work thus extends our understanding of how ORO suppresses gametophyte identity in the diploid phase of the *Ectocarpus* life cycle, which could potentially occur via the direct repression of sex-determining genes. Future *in vivo* binding studies will hopefully further our molecular understanding of how ORO exerts its regulatory role over the U and V sex chromosomes.

Our work further sought to clarify how sexual differentiation programmes are differentially regulated across the *Ectocarpus* life cycle. Altogether, our data suggest that the regulation of SDR genes is largely independent of sRNAs because we found no global association between the presence of sRNAs and transcriptional silencing of SDR genes in the sporophyte generation. One notable exception was the U-specific gene *Ec-sdr­­_f_00040*, which was upregulated in *oro;oro* mutants and was correspondingly correlated with reduced sRNA levels. *Ec-sdr_f_000040* belongs to a highly divergent gametolog pair representing a casein kinase gene that is consistently female-sex linked across a range of brown algal species (Lipinska et al., 2017). Casein kinases are involved in regulating a wide range of developmental processes, including reproduction (Guo et al., 2023; Ogiso et al., 2010; Phadnis et al., 2015; Qu et al., 2021; Zhang et al., 2022), and its orthologue in kelps appears to have an important function in female development (Müller et al., 2021). We speculate that this is gene could also be involved in the transcriptional feminization of *oro;oro* mutants. Future examination will be required to further understand the intriguing accumulation of sRNAs over the repeat element within the intron of this sex-linked gene.

Despite the transcriptional changes we observed at genes involved in sex determination, we were puzzled not to find concomitant changes in chromatin state. The transition to sexual development in *Ectocarpus* is characterized by the formation of plurilocular sporangia that produce and release motile gametes (Charrier et al., 2008; Coelho et al., 2007). SDR genes, as well as other sex-biased genes, are thus only likely to be transcribed within a small subset of reproductive cells and/or during a limited time window of plurilocular sporangia development. Given that our profiles were generated using whole thalli, we speculate that the excess of vegetative tissue could impede our ability to observe cell type-specific changes in chromatin state. Generating such data is challenging owing to the microscopic nature of these reproductive structures in *Ectocarpus*. Analyses at the single-cell level will hopefully clarify how chromatin reprogramming facilitates sexual differentiation during the transition to the haploid gametophyte generation.

Nevertheless, our chromatin profiling has revealed how H3K79me2 deposition is re-configured across the *Ectocarpus* life cycle. In particular, we observed a strong reduction of H3K79me2 along the SDRs in the sporophyte generation, particularly along the female SDR of the U chromosome, which strongly contrasts with its gametophytic pattern (Bourdareau et al., 2021; Farooq et al., 2016; Gueno et al., 2022). H3K79me2 has been extensively studied in yeast and mammals where it accumulates over the transcribed region of active genes (Nguyen and Zhang, 2011). In contrast, H3K79me2 has an opposing pattern in *Ectocarpus* gametophytes as it preferentially accumulates over transposons and genes with low levels of gene expression (Bourdareau et al., 2021; Gueno et al., 2022). Interestingly, we show here that this association is less evident in the sporophyte because its accumulation is no longer associated with obvious changes in gene expression. This suggests that the role of H3K79me2 may be distinct during the diploid phase of the life cycle and might no longer mediate a repressive role.

In the gametophyte-dominant bryophyte *Marchantia*, paternal chromosomes are repressed by H3K27me3 to result in functionally haploid embryos during the reduced diploid sporophytic phase of the life cycle (Montgomery et al., 2022). Whether chromosome inactivation is conserved in the sporophyte of related bryophytes and other haploid-diploid organisms such as macroalgae is unclear. Insufficient genetic variation between the parents of the sporophyte used in our study prevented us from addressing this phenomenon in *Ectocarpus* (Gueno et al., 2022). Nevertheless, we speculate that such phenomena are unlikely to occur given that the *Ectocaprus* sporophyte is not dependent on the female gametophyte as in *Marchantia*, because both the gametophyte and sporophyte are free-living, nearly isomorphic and equally dominant in duration. The reconfiguration of H3K79me2 during the haploid-diploid transition in *Ectocarpus* is also reminiscent of the extensive epigenetic reprogramming observed during haploid-diploid transitions in flowering plants (Vigneau and Borg, 2021), which play a key role in activating pollen-specific genes (Borg et al., 2021; Khouider et al., 2021), sperm specification (Borg et al., 2020) and specification of the female gametophyte in *Arabidopsis* (Baroux and Autran, 2015; She and Baroux, 2015; She et al., 2013). Such chromatin reprogramming events may thus be a general feature of organisms that alternate between haploid and diploid multicellular phases, although future studies are needed to clarify the precise role of H3K79me2 and its potential impact on life cycle transitions in the brown algal lineage.

## MATERIALS AND METHODS

### Biological material

The pedigrees of the strains used in this study were described previously (Coelho et al., 2011) and correspond to WT male (Ec32) and female (Ec25) gametophytes, WT sporophytes (Ec17), *oro* mutant males (Ec561) and females (Ec560), and diploid *oro;oro* mutants (Ec581). About ten algal individuals were cultivated in Petri dishes at 14°C in Provasoli-enriched seawater (Coelho et al., 2012) under a short-day regime (12 h dark/12 h light) with 20 μmol photons m^−2^ s^−1^ irradiance.

### RNA-seq analysis

RNA-seq data were generated from culture with same conditions to ensure that the histone post-translational modification and gene expression data were fully compatible. All RNA-seq datasets generated in this study were carried out in biological triplicate for each genotype. For each replicate, 10 mg of *Ectocarpus* tissue (approximately ten individuals) was patted dry with absorbent tissue paper and flash-frozen. Total RNA was isolated as described previously with minor modifications (Cossard et al., 2022; Lipinska et al., 2015; Wang and Stegemann, 2010). In brief, the tissue was ground to a fine powder in liquid nitrogen in 1.5 ml Eppendorf tubes using a micropestle. The algae powder was further homogenized in 700 μl of pre-warmed (at 65°C) cetlytrimethylammonium bromide (CTAB)-based extraction buffer [100 mM Tris-HCl pH 8, 1.4 M NaCl, 20 mM EDTA pH 8, 2% Plant RNA Isolation Aid (PVP, Invitrogen AM9690), 2% CTAB and 1% β-mercaptoethanol] by vortexing and incubating at 65°C until all samples were processed (5-20 min). RNA was extracted by mixing the homogenate with 1:1 volume of chloroform/isoamylalcohol (24:1). Supernatant was collected after centrifugation at 10,000 ***g*** for 15 min at 4°C and the chloroform/isoamylalcohol extraction step was repeated. RNA was precipitated with 3 M LiCl and 1% (v/v) β-mercaptoethanol at −20°C overnight. Samples were centrifuged at 20,000 ***g*** for 1 h at 4°C. Pellets were washed with ice-cold 70% ethanol and then dissolved in RNase-free water. DNase treatment was performed using a TURBO DNase Kit according to the manufacturer's instructions (Thermo Fisher Scientific, AM1907). Libraries were constructed with the NEBNext^®^ Ultra™ II Directional RNA Library Prep Kit (New England Biolabs, E7760S) and sequenced on an Illumina NextSeq2000 platform to generate a minimum of 25-30 million 150-bp paired-end reads per sample.

The RNA-seq samples were processed according to the methods described by Perroud et al. (2018) and updated (Haas et al., 2020). The software packages used by the RNA-seq pipeline were updated to the most recent versions: gmap-gsnap (Wu and Nacu, 2010) version 2021-12-17; samtools (Li et al., 2009) version 1.15.1. Read count software was exchanged by subread featurecounts (Liao et al., 2014), version 2.0.3. Published RNA-seq datasets for WT male and female gametophytes were downloaded and processed in the same manner (Gueno et al., 2022; NCBI BioProject PRJNA671807). Differential gene expression analysis was performed using DESeq2 version 1.30.1 (Love et al., 2014). DEGs were classified as having a log_2_ fold-change difference of 1 and a *P-*value of <0.05 after correction for multiple testing (i.e. an adjusted *P-*value). We considered a conservative set of 94 SBGs that were commonly sex-biased (i.e. differentially expressed in WT male or WT female gametophytes) in at least two out of three independent public datasets (Table S2). Male and female SDR genes, gametologs and PAR genes were described previously (Gueno et al., 2022). RNA-seq heatmaps were generated using the pheatmap package in R (https://github.com/raivokolde/pheatmap). Violin plots were generated using the ggplot2 package in R (https://ggplot2.tidyverse.org) and dot plots were generated using base R.

### Small RNA analysis

WT sporophyte (Ec17) and *oro;oro* mutant (Ec581) tissue was grown and frozen as described in the ‘RNA-seq analysis’ section. For total RNA isolation, 50 mg of tissue was used, employing a modified version of the CTAB-based method described above. One millilitre of CTAB buffer was used for homogenization, and precipitation was carried out with a 1:1 volume of iso-propanol at −20°C overnight to preserve small RNA molecules. DNase treatment was performed using a TURBO DNase Kit according to the manufacturer's instructions (Thermo Fisher Scientific, AM1907). Total RNA was purified with RNA clean and concentrator columns (Zymo Research, R1013) following the manufacturer's instructions with important modifications to the washing steps. Columns were washed twice with 400 μl of RNA Prep buffer and four times with 700 μl of RNA Wash Buffer. Library preparation and sequencing were carried out by Novogene. Briefly, 5′ and 3′ adaptors were ligated to small RNA ends followed by first-strand cDNA synthesis after hybridization with a reverse transcription primer. Double-stranded cDNA libraries were generated by PCR enrichment. Fragments containing inserts between ∼18 and 40 bp were size selected and purified prior to Illumina sequencing to generate 50-bp single-end reads.

Data quality control, trimming and mapping were performed with a Snakemake sRNA pipeline (https://github.com/seb-mueller/snakemake_sRNAseq). In brief, FastQC (v0.11.7) was used to assess read quality, followed by 3′ adaptor removal using cutadapt to trim Illumina universal adapters. All sequences <18 bp and >40 bp in length were filtered and the remaining sequences mapped to the *Ectocarpus sp.* 7 reference genome (https://phaeoexplorer.sb-roscoff.fr/public/organism/ectocarpus-sp7/). Mapping was performed using Bowtie version 1.2 with no mismatches allowed (Langmead and Salzberg, 2012). Both unique and multi-mapped sRNAs were considered for downstream analysis. The config.yaml files used for this analysis can be accessed at https://doi.org/10.17617/3.4SCUJN. sRNA quantity was normalized as count per million (CPM) and counted over gene models using the ‘featureCounts’ tool in the R package Subread with default parameters (Liao et al., 2014). Plots and statistical tests were performed with base R and ggplot2 1.0. DESeq2 was used to calculate differential sRNA accumulation (Love et al., 2014). Correlation analysis was carried out using the ‘ggscatter’ function (add=“reg.line”, conf.int=TRUE, cor.coef=TRUE, cor.method=“spearman”) with the R package Ggpubr (https://cran.r-project.org/web/packages/ggpubr/index.html).

### ChIP-seq analysis

ChIP-seq profiling of H3K4me3, H3K9ac, H4K20me3 and H3K79me2 were performed as described previously (Bourdareau et al., 2022). In brief, approximately 1 g of semi-dry *Ectocarpus* tissue (corresponding to around 1000 individuals) was fixed in seawater containing 1% formaldehyde for 5 min. Cross-formaldehyde was eliminated by washing with fresh seawater and the cross-linking quenched by incubation in 1× PBS containing 400 mM glycine for 5 min. Nuclei were isolated by grinding the cross-linked tissue in liquid nitrogen, resuspended in nuclear isolation buffer [0.1% Triton X-100, 125 mM sorbitol, 20 mM potassium citrate, 30 mM MgCl_2_, 5 mM EDTA, 5 mM β-mercaptoethanol, 55 mM HEPES at pH 7.5, 1× cOmplete protease inhibitor cocktails (Roche)], then gently ground in a Tenbroeck Potter. The suspension was filtered through Miracloth then centrifuged at 3000 ***g*** for 20 min to pellet the nuclei. The nuclear pellets were washed twice with fresh nuclear isolation buffer and once with nuclear isolation buffer containing no Triton X-100. The final pellet was resuspended in 750 μl nuclear lysis buffer [10 mM EDTA, 1% SDS, 50 mM Tris-HCl at pH 8, 1× cOmplete protease inhibitor cocktails (Roche)]. Chromatin was fragmented by sonicating the nuclear suspension in a microTUBE AFA Fiber Snap-Cap 6×16 mm using a Covaris E220 Evolution sonicator (duty 25%, peak power 75, cycles/burst 200, duration 900 s at 4°C). The sonicated suspension was then centrifuged at 14,000 ***g*** for 5 min at 4°C to remove cell debris. The supernatant containing fragmented chromatin was collected and diluted ten times with ChIP dilution buffer [1% Triton X-100, 1.2 mM EDTA, 16.7 mM Tris-HCl pH 8, 167 mM NaCl and 1× cOmplete protease inhibitor cocktail (Roche)]. The chromatin solution was split among multiple DNA-Lo Bind Tubes (Eppendorf) and incubated with antibodies on a rotator at 10 rpm overnight at 4°C. All histone antibodies were purchased from Cell Signaling Technology (anti-histone H3, 4620; anti-H3K4me3, 9751S; anti-H3K9ac, 9649S; anti-H3K79me2, D15E8; anti-H4K20me3, 5737S). Immunoprecipitation was performed using an equal mix of protein A and protein G Dynabeads (Thermo Fisher Scientific, 10004D and 10002D). Following immunoprecipitation and washing steps, samples were eluted in 100 μl Direct Elution Buffer (0.5% SDS, 5 mM EDTA, 10 mM Tris-HCl pH 8, 300 mM NaCl). Cross-links were reversed by incubating the samples at 65°C overnight with intermittent shaking. The samples were digested with Proteinase K (Fisher Scientific, 11826724) and RNase A (Roche, 10109142001) prior to DNA purification using AMPure beads (Beckman Coulter, A63882). Libraries were constructed with the NEBNext^®^ Ultra™ II DNA Library Prep Kit (New England Biolabs, E7645S) and sequenced on an Illumina HiSeq 3000 platform to generate a minimum of 20 million 150-bp reads per sample.

Two biological replicates of each genotype were mapped onto the *Ectocarpus sp.* 7 reference genome (https://phaeoexplorer.sb-roscoff.fr/public/organism/ectocarpus-sp7/) using the Nextflow nf-core/chipseq pipeline v1.2.2 (Harshil et al., 2021). Published ChIP-Seq datasets for WT male and female gametophytes were downloaded and processed in the same manner (Gueno et al., 2022). A MultiQC report (Ewels et al., 2016) of each run with quality control metrics of each dataset, including trimming, mapping, coverage and complexity metrics, as well as the version of each tool used in the pipeline, can be accessed at https://doi.org/10.17617/3.4SCUJN. For data visualization and plotting, normalized log_2_ bigwig coverage files of each histone mark relative to H3 were generated using deepTools version 3.5.1 bamCompare with a bin size of 10 bp. Biological replicates were merged for downstream analysis after confirming high correlation (Fig. S2). Cross-correlation matrices of Spearman's correlation coefficient were generated by comparing log_2_ coverage relative to H3 using deepTools version 3.5.1 multiBigwigSummary (Ramirez et al., 2014). Bigwig coverage files were visualized along the *Ectocarpus* genome using IGV version 2.16.2 (Robinson et al., 2011). Averaged ChIP-Seq profiles were generated using the R package EnrichedHeatmap normalizeToMatrix (Gu et al., 2018) and plotted using a custom script or with deepTools version 3.5.1 plotProfile (Ramirez et al., 2014). Differential analysis of H3K79me2 peaks between WT gametophytes and sporophytes was performed using the R package DiffBind version 3.18 (https://bioconductor.org/packages/release/bioc/html/DiffBind.html). Hierarchical clustering of H3K79me2 profiles among the different genotypes was computed with deepTools version 3.5.1 computeMatrix using log_2_ H3K79me2 coverage relative to H3.

## Supplementary Material



10.1242/develop.202677_sup1 Supplementary information

Table S1. TPM values of RNA-seq data used in this study alongside differential expression data of comparisons between WT and oro gametophytes.

Table S2. TPM values of the sex-biased genes used in this study.

Table S3. Differentially-expressed transcripts and associated sRNAs at SDR genes.

Table S4. H3K79me2 peaks with significant differential enrichment between WT sporophyte and WT female gametophytes.

Table S5. H3K79me2 peaks with significant differential enrichment between WT sporophyte and WT male gametophytes.

## References

[DEV202677C1] Ahmed, S., Cock, J. M., Pessia, E., Luthringer, R., Cormier, A., Robuchon, M., Sterck, L., Peters, A. F., Dittami, S. M., Corre, E. et al. (2014). A haploid system of sex determination in the brown alga *Ectocarpus* sp. *Curr. Biol.* 24, 1945-1957. 10.1016/j.cub.2014.07.042 25176635

[DEV202677C2] Allen, S. L., Bonduriansky, R. and Chenoweth, S. F. (2018). Genetic constraints on microevolutionary divergence of sex-biased gene expression. *Philos. Trans. R. Soc. B Biol. Sci.* 373, 20170427. 10.1098/rstb.2017.0427 PMC6125734 30150225

[DEV202677C3] Arun, A., Coelho, S. M., Peters, A. F., Bourdareau, S., Peres, L., Scornet, D., Strittmatter, M., Lipinska, A. P., Yao, H., Godfroy, O. et al. (2019). Convergent recruitment of TALE homeodomain life cycle regulators to direct sporophyte development in land plants and brown algae. *eLife* 8, e43101. 10.7554/eLife.43101 30644818 PMC6368402

[DEV202677C4] Avia, K., Lipinska, A. P., Mignerot, L., Montecinos, A. E., Jamy, M., Ahmed, S., Valero, M., Peters, A. F., Cock, J. M., Roze, D. et al. (2018). Genetic diversity in the UV sex chromosomes of the brown alga *Ectocarpus*. *Genes* 9, 286. 10.3390/genes9060286 29882839 PMC6027523

[DEV202677C5] Bachtrog, D. (2006). A dynamic view of sex chromosome evolution. *Curr. Opin. Genet. Dev.* 16, 578-585. 10.1016/j.gde.2006.10.007 17055249

[DEV202677C6] Baroux, C. and Autran, D. (2015). Chromatin dynamics during cellular differentiation in the female reproductive lineage of flowering plants. *Plant J. Cell Mol. Biol.* 83, 160-176. 10.1111/tpj.12890 PMC4502977 26031902

[DEV202677C7] Borg, M., Jacob, Y., Susaki, D., LeBlanc, C., Buendía, D., Axelsson, E., Kawashima, T., Voigt, P., Boavida, L., Becker, J. et al. (2020). Targeted reprogramming of H3K27me3 resets epigenetic memory in plant paternal chromatin. *Nat. Cell Biol.* 22, 621-629. 10.1038/s41556-020-0515-y 32393884 PMC7116658

[DEV202677C8] Borg, M., Papareddy, R. K., Dombey, R., Axelsson, E., Nodine, M. D., Twell, D. and Berger, F. (2021). Epigenetic reprogramming rewires transcription during the alternation of generations in Arabidopsis. *eLife* 10, e61894. 10.7554/eLife.61894 33491647 PMC7920552

[DEV202677C9] Bothwell, J. H., Marie, D., Peters, A. F., Cock, J. M. and Coelho, S. M. (2010). Cell cycles and endocycles in the model brown seaweed, *Ectocarpus siliculosus*. *Plant Signal. Behav.* 5, 1473-1475. 10.4161/psb.5.11.13520 21057192 PMC3115259

[DEV202677C10] Bourdareau, S., Tirichine, L., Lombard, B., Loew, D., Scornet, D., Wu, Y., Coelho, S. M. and Cock, J. M. (2021). Histone modifications during the life cycle of the brown alga Ectocarpus. *Genome Biol.* 22, 12. 10.1186/s13059-020-02216-8 33397407 PMC7784034

[DEV202677C11] Bourdareau, S., Godfroy, O., Gueno, J., Scornet, D., Coelho, S. M., Tirichine, L. and Cock, J. M. (2022). An Efficient Chromatin Immunoprecipitation Protocol for the Analysis of Histone Modification Distributions in the Brown Alga Ectocarpus. *Methods Protoc.* 5, 36. 10.3390/mps5030036 35645344 PMC9149930

[DEV202677C12] Brown, E. J. and Bachtrog, D. (2014). The chromatin landscape of Drosophila: comparisons between species, sexes, and chromosomes. *Genome Res.* 24, 1125-1137. 10.1101/gr.172155.114 24840603 PMC4079968

[DEV202677C13] Bull, J. J. (1978). Sex chromosomes in haploid dioecy: a unique contrast to Muller's theory for diploid dioecy. *Am. Nat.* 112, 245-250. 10.1086/283267

[DEV202677C14] Carey, S. B., Jenkins, J., Lovell, J. T., Maumus, F., Sreedasyam, A., Payton, A. C., Shu, S., Tiley, G. P., Fernandez-Pozo, N., Healey, A. et al. (2021). Gene-rich UV sex chromosomes harbor conserved regulators of sexual development. *Sci. Adv.* 7, eabh2488. 10.1126/sciadv.abh2488 34193417 PMC8245031

[DEV202677C15] Charlesworth, D. (2016). Plant sex chromosomes. *Annu. Rev. Plant Biol.* 67, 397-420. 10.1146/annurev-arplant-043015-111911 26653795

[DEV202677C16] Charrier, B., Coelho, S. M., Le Bail, A., Tonon, T., Michel, G., Potin, P., Kloareg, B., Boyen, C., Peters, A. F. and Cock, J. M. (2008). Development and physiology of the brown alga *Ectocarpus siliculosus*: two centuries of research. *New Phytol.* 177, 319-332. 10.1111/j.1469-8137.2007.02304.x 18181960

[DEV202677C17] Chen, X. (2009). Small RNAs and their roles in plant development. *Annu. Rev. Cell Dev. Biol.* 25, 21-44. 10.1146/annurev.cellbio.042308.113417 19575669 PMC5135726

[DEV202677C18] Coelho, S. M. and Umen, J. (2021). Switching it up: algal insights into sexual transitions. *Plant Reprod.* 34, 287-296. 10.1007/s00497-021-00417-0 34181073 PMC8566403

[DEV202677C19] Coelho, S. M., Peters, A. F., Charrier, B., Roze, D., Destombe, C., Valero, M. and Cock, J. M. (2007). Complex life cycles of multicellular eukaryotes: new approaches based on the use of model organisms. *Gene* 406, 152-170. 10.1016/j.gene.2007.07.025 17870254

[DEV202677C20] Coelho, S. M., Godfroy, O., Arun, A., Le Corguillé, G., Peters, A. F. and Cock, J. M. (2011). OUROBOROS is a master regulator of the gametophyte to sporophyte life cycle transition in the brown alga *Ectocarpus*. *Proc. Natl. Acad. Sci. USA* 108, 11518-11523. 10.1073/pnas.1102274108 21709217 PMC3136289

[DEV202677C21] Coelho, S. M., Scornet, D., Rousvoal, S., Peters, N. T., Dartevelle, L., Peters, A. F. and Cock, J. M. (2012). How to cultivate *Ectocarpus*. *Cold Spring Harb. Protoc.* 2012, 258-261. 22301662 10.1101/pdb.prot067934

[DEV202677C22] Coelho, S. M., Gueno, J., Lipinska, A. P., Cock, J. M. and Umen, J. G. (2018). UV chromosomes and haploid sexual systems. *Trends Plant Sci.* 23, 794-807. 10.1016/j.tplants.2018.06.005 30007571 PMC6128410

[DEV202677C23] Coelho, S. M., Mignerot, L. and Cock, J. M. (2019). Origin and evolution of sex-determination systems in the brown algae. *New Phytol.* 222, 1751-1756. 10.1111/nph.15694 30667071

[DEV202677C24] Cossard, G. G., Godfroy, O., Nehr, Z., Cruaud, C., Cock, J. M., Lipinska, A. P. and Coelho, S. M. (2022). Selection drives convergent gene expression changes during transitions to co-sexuality in haploid sexual systems. *Nat. Ecol. Evol.* 6, 579-589. 10.1038/s41559-022-01692-4 35314785 PMC9085613

[DEV202677C25] Dierschke, T., Flores-Sandoval, E., Rast-Somssich, M. I., Althoff, F., Zachgo, S. and Bowman, J. L. (2021). Gamete expression of TALE class HD genes activates the diploid sporophyte program in Marchantia polymorpha. *eLife* 10, e57088. 10.7554/eLife.57088 34533136 PMC8476127

[DEV202677C26] Disteche, C. M. (2012). Dosage compensation of the sex chromosomes. *Annu. Rev. Genet.* 46, 537-560. 10.1146/annurev-genet-110711-155454 22974302 PMC3767307

[DEV202677C27] Duan, J. and Larschan, E. N. (2019). Dosage compensation: how to be compensated…or not? *Curr. Biol.* 29, R1229-R1231. 10.1016/j.cub.2019.09.065 31794753 PMC8897756

[DEV202677C28] Ewels, P., Magnusson, M., Lundin, S. and Käller, M. (2016). MultiQC: summarize analysis results for multiple tools and samples in a single report. *Bioinformatics* 32, 3047-3048. 10.1093/bioinformatics/btw354 27312411 PMC5039924

[DEV202677C29] Farooq, Z., Banday, S., Pandita, T. K. and Altaf, M. (2016). The many faces of histone H3K79 methylation. *Mutat. Res. Mutat. Res.* 768, 46-52. 10.1016/j.mrrev.2016.03.005 PMC4889126 27234562

[DEV202677C30] Gu, Z., Eils, R., Schlesner, M. and Ishaque, N. (2018). EnrichedHeatmap: an R/Bioconductor package for comprehensive visualization of genomic signal associations. *BMC Genomics* 19, 234. 10.1186/s12864-018-4625-x 29618320 PMC5885322

[DEV202677C31] Gueno, J., Borg, M., Bourdareau, S., Cossard, G., Godfroy, O., Lipinska, A., Tirichine, L., Cock, J. M. and Coelho, S. M. (2022). Chromatin landscape associated with sexual differentiation in a UV sex determination system. *Nucleic Acids Res.* 50, 3307-3322. 10.1093/nar/gkac145 35253891 PMC8989524

[DEV202677C32] Guo, H., Zhang, D., Zhou, Y., Sun, L., Li, C., Luo, X., Liu, J. and Cui, S. (2023). Casein kinase 1α regulates testosterone synthesis and testis development in adult mice. *Endocrinology* 164, bqad042. 10.1210/endocr/bqad042 36929849

[DEV202677C33] Haas, F. B., Fernandez-Pozo, N., Meyberg, R., Perroud, P.-F., Göttig, M., Stingl, N., Saint-Marcoux, D., Langdale, J. A. and Rensing, S. A. (2020). Single nucleotide polymorphism charting of P. patens reveals accumulation of somatic mutations during in vitro culture on the scale of natural variation by selfing. *Front. Plant Sci.* 11, 813. 10.3389/fpls.2020.00813 32733496 PMC7358436

[DEV202677C34] Patel, H., Wang, C., Ewels, P., Chedraoui Silva, T., Peltzer, A., Behrens, D., Garcia, M., mashehu, Rotholandus, Haglund, S. and Kretzschmar, W. (2021). nf-core/chipseq: nf-core/chipseq v1.2.2 - Rusty Mole. 10.5281/zenodo.4711243

[DEV202677C35] Kariyawasam, T., Joo, S., Lee, J., Toor, D., Gao, A. F., Noh, K.-C. and Lee, J.-H. (2019). TALE homeobox heterodimer GSM1/GSP1 is a molecular switch that prevents unwarranted genetic recombination in Chlamydomonas. *Plant J. Cell Mol. Biol.* 100, 938-953. 10.1111/tpj.14486 31368133

[DEV202677C36] Khouider, S., Borges, F., LeBlanc, C., Ungru, A., Schnittger, A., Martienssen, R., Colot, V. and Bouyer, D. (2021). Male fertility in Arabidopsis requires active DNA demethylation of genes that control pollen tube function. *Nat. Commun.* 12, 410. 10.1038/s41467-020-20606-1 33462227 PMC7813888

[DEV202677C37] Langmead, B. and Salzberg, S. L. (2012). Fast gapped-read alignment with Bowtie 2. *Nat. Methods* 9, 357-359. 10.1038/nmeth.1923 22388286 PMC3322381

[DEV202677C38] Li, H., Handsaker, B., Wysoker, A., Fennell, T., Ruan, J., Homer, N., Marth, G., Abecasis, G. and Durbin, R. and 1000 Genome Project Data Processing Subgroup. (2009). The Sequence Alignment/Map format and SAMtools. *Bioinformatics* 25, 2078-2079. 10.1093/bioinformatics/btp352 19505943 PMC2723002

[DEV202677C39] Liao, Y., Smyth, G. K. and Shi, W. (2014). featureCounts: an efficient general purpose program for assigning sequence reads to genomic features. *Bioinformatics* 30, 923-930. 10.1093/bioinformatics/btt656 24227677

[DEV202677C40] Lipinska, A. P., Cormier, A., Luthringer, R., Peters, A. F., Corre, E., Gachon, C. M. M., Cock, J. M. and Coelho, S. M. (2015). Sexual dimorphism and the evolution of sex-biased gene expression in the brown alga *Ectocarpus*. *Mol. Biol. Evol.* 32, 1581-1597. 10.1093/molbev/msv049 25725430

[DEV202677C41] Lipinska, A. P., Toda, N. R. T., Heesch, S., Peters, A. F., Cock, J. M. and Coelho, S. M. (2017). Multiple gene movements into and out of haploid sex chromosomes. *Genome Biol.* 18, 104. 10.1186/s13059-017-1201-7 28595587 PMC5463336

[DEV202677C42] Love, M. I., Huber, W. and Anders, S. (2014). Moderated estimation of fold change and dispersion for RNA-seq data with DESeq2. *Genome Biol.* 15, 550. 10.1186/s13059-014-0550-8 25516281 PMC4302049

[DEV202677C43] Luthringer, R., Cormier, A., Peters, A. F., Cock, J. M. and Coelho, S. M. and Coelho, S. M. (2015a). Sexual dimorphism in the brown algae. *Perspect. Phycol.* 1, 11-25. 10.1127/2198-011X/2014/0002

[DEV202677C44] Luthringer, R., Lipinska, A. P., Roze, D., Cormier, A., Macaisne, N., Peters, A. F., Cock, J. M. and Coelho, S. M. (2015b). The pseudoautosomal regions of the U/V sex chromosomes of the brown alga *Ectocarpus* exhibit unusual features. *Mol. Biol. Evol.* 32, 2973-2985. 10.1093/molbev/msv173 26248564 PMC4610043

[DEV202677C45] Luthringer, R., Raphalen, M., Guerra, C., Colin, S., Martinho, C., Zheng, M., Hoshino, M., Badis, Y., Lipinska, A. P., Haas, F. B. et al. (2024). Repeated co-option of HMG-box genes for sex determination in brown algae and animals. *Science*. 383, eadk5466. 10.1126/science.adk5466 38513029

[DEV202677C46] Maier, I. and Muller, D. G. (1986). Sexual pheromones in algae. *The Biological Bulletin*, 170, 145-175.

[DEV202677C47] Meller, V. H. (2000). Dosage compensation: making 1X equal 2X. *Trends Cell Biol.* 10, 54-59. 10.1016/S0962-8924(99)01693-1 10652515

[DEV202677C48] Montgomery, S. A., Hisanaga, T., Wang, N., Axelsson, E., Akimcheva, S., Sramek, M., Liu, C. and Berger, F. (2022). Polycomb-mediated repression of paternal chromosomes maintains haploid dosage in diploid embryos of Marchantia. *eLife* 11, e79258. 10.7554/eLife.79258 35996955 PMC9402228

[DEV202677C49] Müller, D. G. (1967). Generationswechsel, Kernphasenwechsel und Sexualität der Braunalge *Ectocarpus siliculosus* im Kulturversuch. *Planta* 75, 39-54. 10.1007/BF00380838 24550014

[DEV202677C50] Müller, D. G., Gaschet, E., Godfroy, O., Gueno, J., Cossard, G., Kunert, M., Peters, A. F., Westermeier, R., Boland, W., Cock, J. M. et al. (2021). A partially sex–reversed giant kelp sheds light into the mechanisms of sexual differentiation in a UV sexual system. *New Phytol.* 232, 252-263. 10.1111/nph.17582 34166525

[DEV202677C51] Nguyen, A. T. and Zhang, Y. (2011). The diverse functions of Dot1 and H3K79 methylation. *Genes Dev.* 25, 1345-1358. 10.1101/gad.2057811 21724828 PMC3134078

[DEV202677C52] Ogiso, E., Takahashi, Y., Sasaki, T., Yano, M. and Izawa, T. (2010). The role of Casein kinase II in flowering time regulation has diversified during evolution. *Plant Physiol.* 152, 808-820. 10.1104/pp.109.148908 20007447 PMC2815897

[DEV202677C53] Perrin, N. (2012). What uses are mating types? The ‘developmental switch’ model. *Evolution* 66, 947-956. 10.1111/j.1558-5646.2011.01562.x 22486681

[DEV202677C54] Perroud, P., Haas, F. B., Hiss, M., Ullrich, K. K., Alboresi, A., Amirebrahimi, M., Barry, K., Bassi, R., Bonhomme, S., Chen, H. et al. (2018). The *Physcomitrella patens* gene atlas project: large-scale RNA-seq based expression data. *Plant J.* 95, 168-182. 10.1111/tpj.13940 29681058

[DEV202677C55] Phadnis, N., Cipak, L., Polakova, S., Hyppa, R. W., Cipakova, I., Anrather, D., Karvaiova, L., Mechtler, K., Smith, G. R. and Gregan, J. (2015). Casein Kinase 1 and phosphorylation of Cohesin Subunit Rec11 (SA3) promote meiotic recombination through linear element formation. *PLoS Genet.* 11, e1005225. 10.1371/journal.pgen.1005225 25993311 PMC4439085

[DEV202677C56] Qu, L., Wei, Z., Chen, H.-H., Liu, T., Liao, K. and Xue, H.-W. (2021). Plant casein kinases phosphorylate and destabilize a cyclin-dependent kinase inhibitor to promote cell division. *Plant Physiol.* 187, 917-930. 10.1093/plphys/kiab284 34608955 PMC8491028

[DEV202677C57] Ramirez, F., Dundar, F., Diehl, S., Gruning, B. A. and Manke, T. (2014). deepTools: a flexible platform for exploring deep-sequencing data. *Nucleic Acids Res.* 42, W187-W191. 10.1093/nar/gku365 24799436 PMC4086134

[DEV202677C58] Rana, T. M. (2007). Illuminating the silence: understanding the structure and function of small RNAs. *Nat. Rev. Mol. Cell Biol.* 8, 23-36. 10.1038/nrm2085 17183358

[DEV202677C59] Robinson, J. T., Thorvaldsdóttir, H., Winckler, W., Guttman, M., Lander, E. S., Getz, G. and Mesirov, J. P. (2011). Integrative genomics viewer. *Nat. Biotechnol.* 29, 24-26. 10.1038/nbt.1754 21221095 PMC3346182

[DEV202677C60] She, W. and Baroux, C. (2015). Chromatin dynamics in pollen mother cells underpin a common scenario at the somatic-to-reproductive fate transition of both the male and female lineages in Arabidopsis. *Front. Plant Sci.* 6, 294. 10.3389/fpls.2015.00294 25972887 PMC4411972

[DEV202677C61] She, W., Grimanelli, D., Rutowicz, K., Whitehead, M. W. J., Puzio, M., Kotliński, M., Jerzmanowski, A. and Baroux, C. (2013). Chromatin reprogramming during the somatic-to-reproductive cell fate transition in plants. *Development* 140, 4008-4019. 10.1242/dev.095034 24004947

[DEV202677C62] Vigneau, J. and Borg, M. (2021). The epigenetic origin of life history transitions in plants and algae. *Plant Reprod.* 34, 267-285. 10.1007/s00497-021-00422-3 34236522 PMC8566409

[DEV202677C63] Wang, L. and Stegemann, J. P. (2010). Extraction of high quality RNA from polysaccharide matrices using cetlytrimethylammonium bromide. *Biomaterials* 31, 1612-1618. 10.1016/j.biomaterials.2009.11.024 19962190 PMC2813910

[DEV202677C64] Wu, T. D. and Nacu, S. (2010). Fast and SNP-tolerant detection of complex variants and splicing in short reads. *Bioinformatics* 26, 873-881. 10.1093/bioinformatics/btq057 20147302 PMC2844994

[DEV202677C65] Zhang, D., Jiang, Y., Luo, X., Liu, H., Zhou, Y. and Cui, S. (2022). Oocyte Casein kinase 1α deletion causes defects in primordial follicle formation and oocyte loss by impairing oocyte meiosis and enhancing autophagy in developing mouse ovary. *Cell Death Discov.* 8, 388. 10.1038/s41420-022-01184-1 36115846 PMC9482644

