# Supplementary material for: Interactions between U and V sex chromosomes during the life cycle of Ectocarpus
Source: Development. 2024 Apr 12;151(20):dev202677. doi: 10.1242/dev.202677 (PMC11057875; doi:10.1242/dev.202677)
Supplement: Supplementary information [file develop-151-202677-s1.pdf]

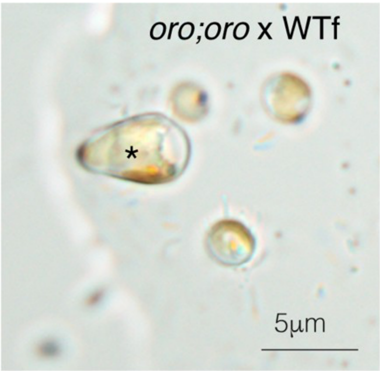

Fig. S1. ***oro;oro*** **mutants function as fertile male gametophytes.** Image of a developing zygote (asterisk) obtained from a cross between a wild type (WT) female and diploid *oro;oro* mutant strain.

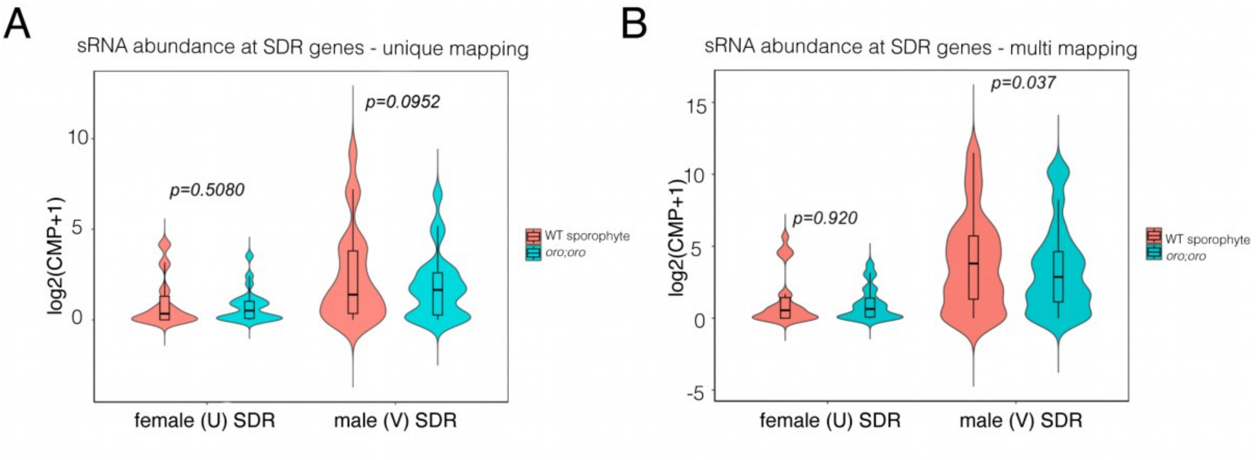

Fig. S2. Small RNA accumulation over genes involved in sex determination. Violin plots summarising the aduncance of uniquely mapped (A) or multi-mapped (B) sRNAs on SDR genes and uniquely mapped (C) or multi-mapped (D) sRNAs on sex-biased genes. U = genes on the SDR of the female U sex chromosome, V = genes on the SDR of the male V sex chromosome, FBG = Female-biased genes, MBG = Male-biased genes. Abundance in the violin plots represents the log_2 of the mean sRNA-seq CPM+1 values. *P* values were computed using a paired Wilcoxon signed-rank test.

Development • Supplementary information

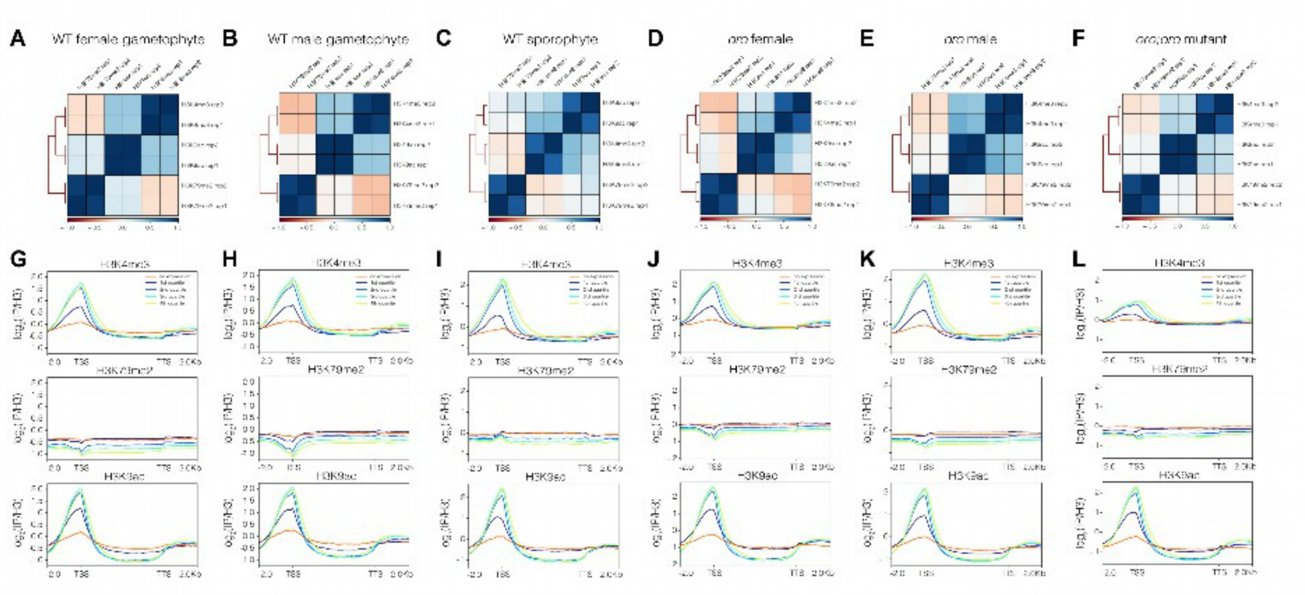

Fig. S3. Quality control of the ChIP-seq datasets generated and/or analysed in this study. Pearson correlation matrices of the ChIP-seq replicates (A-F) and the ChIP-seq signal of H3K4me3, H3K9ac and H3K79me2 over genes sorted by the expression level in each corresponding genotype (G-L). (A,G) WT female gametophyte, (B,H) WT male gametophyte, (C,I) WT sporophyte, (D,J) *oro* females, (E,K) *oro* males and (F,L) diploid *oro;oro* mutants.

Table S1. TPM values of RNA-seq data used in this study alongside differential expression data of comparisons between WT and oro gametophytes.

Available for download at
https://journals.biologists.com/dev/article-lookup/doi/10.1242/dev.202677#supplementary-data

Table S2. TPM values of the sex-biased genes used in this study.

Available for download at
https://journals.biologists.com/dev/article-lookup/doi/10.1242/dev.202677#supplementary-data

Table S3. Differentially-expressed transcripts and associated sRNAs at SDR genes.

Available for download at
https://journals.biologists.com/dev/article-lookup/doi/10.1242/dev.202677#supplementary-data

Table S4. H3K79me2 peaks with significant differential enrichment between WT sporophyte and WT female gametophytes.

Available for download at
https://journals.biologists.com/dev/article-lookup/doi/10.1242/dev.202677#supplementary-data

Table S5. H3K79me2 peaks with significant differential enrichment between WT sporophyte and WT male gametophytes.

Available for download at
https://journals.biologists.com/dev/article-lookup/doi/10.1242/dev.202677#supplementary-data
