# Supplementary material for: Interactions between U and V sex chromosomes during the life cycle of Ectocarpus
Source: Development. 2024 Apr 12;151(20):dev202677. doi: 10.1242/dev.202677 (PMC11057875; doi:10.1242/dev.202677)
Supplement: Supplementary file 1 [file develop-151-202677_review_history.pdf]

Interactions between U and V sex chromosomes during the life cycle of *Ectocarpus*

Jeromine Vigneau, Claudia Martinho, Olivier Godfroy, Min Zheng, Fabian B. Haas, Michael Borg and Susana M. Coelho

DOI: 10.1242/dev.202677

Editor: Liam Dolan

Review timeline

Original submission:	4 January 2024
Editorial decision:	12 February 2024
First revision received:	27 February 2024
Accepted:	1 March 2024

Original submission

First decision letter

MS ID#: DEVELOP/2024/202677

MS TITLE: Sex chromosome dominance in a UV sexual system

AUTHORS: Jeromine Vigneau, Claudia Martinho, Olivier Godfroy, Min Zheg, Fabia Haas, Michael Borg, and Susana M. Coelho

I have now received all the referees reports on the above manuscript, and have reached a decision. The referees' comments are appended below, or you can access them online: please go to BenchPress and click on the 'Manuscripts with Decisions' queue in the Author Area.

The overall evaluation is positive and we would like to publish a revised manuscript in Development, provided that the referees' comments can be satisfactorily addressed. Both reviewers have made recommended changes that will improve your manuscript. However, none has requested more data. Please attend to all of the reviewers' comments in your revised manuscript and detail them in your point-by-point response. If you do not agree with any of their criticisms or suggestions explain clearly why this is so. If it would be helpful, you are welcome to contact us to discuss your revision in greater detail. Please send us a point-by-point response indicating your plans for addressing the referees’ comments, and we will look over this and provide further guidance.

Reviewer 1

Advance summary and potential significance to field

This ms includes some interesting results (see below). I am not qualified to evaluate the experiments on epigenetic modifications, which relate to the mechanisms controlling gene expression, but I believe that they will interest readers of this journal. Some of the other results are intriguing, albeit not always interpreted in relation to overall questions or general concepts. I also think that the material should be organized better.

© 2024. Published by The Company of Biologists under the terms of the Creative Commons Attribution License (https://creativecommons.org/licenses/by/4.0/).

The present text is too repetitive and the English also needs to be improved and the writing shortened. I indicate text that is unnecessary by striking it through in the pdf file, and other text needing corrections is highlighted in purple.

Comments for the author

Reviewer 1 Comments for the Author:
Comments were uploaded and are available as a PDF at https://submit-dev.biologists.org.

Reviewer 2

Advance summary and potential significance to field

Vigneau and co-authors reported the genetic interactions between the U and V chromosomes by analyzing transcription profile and chromatin status across the life cycle of the unique model brown algae Ectocarpus. They also analyzed those of the oro mutant that decouple developmental programs from ploidy. They revealed developmental regulation of the genes encoded in the sex-determining loci of the sex chromosomes. They provided convincing epigenetic data to compare the chromatin status of sexual phenotype of each generation of the life cycle. Overall, the study provided insights for understanding the genetic regulation of the UV sex chromosomes. Several suggestions are listed below for possible improvement of the manuscript.

Comments for the author

Sex chromosome dominance is a fundamental question in biology. However, the analysis of the oro:oro mutant is suggestive but not sufficient to conclude the sex chromosome dominance of the UV system in Ectocarpos. ORO is primarily required for proper sporophyte development (substrate adherence structure) since diploid oro;oro mutants are more closely related to haploid gametophytes. Although male sex determination was observed in oro;oro mutants, it is hard to conclude if the observed male phenotype is essentially due to the dominant character of V over U by oro mutation phenotype or by just independent from oro. What is the default sex of Ectocarpos? The observed negative correlation in transcript accumulation between ORO-SAM and HMG-sex might provide insight, but it is not strong evidence for the conclusion. Ideally, sex chromosome dominance in a UV system would be analyzed in aneuploid isolates with the U and V chromosomes in the gametophyte generation. Therefore, sex chromosome dominance should be still discussed carefully.
Related to the comments above, The paper's concise title "Sex chromosome dominance in a UV sexual system," does not adequately reflect the results shown in the manuscript.
Another reason that the title should be more informative is that this is an original research article, not a review article.

The abstract should also be improved to describe the background information accurately.
The first three sentences broadly state the importance of the subject of the study; the first and third sentences are correct, but the second sentence that starts with "In such organisms" is incorrect. Vascular plants have an alternation of generations, but most of them do not have sex chromosomes, and when they do, sex chromosomes are XX/XY (diploid system), not U and V. The sex-determination system by UV sex chromosomes needs to be accurately described so that the general readers should easily understand the differences from well-known XX/XY (ZW/ZZ) without confusion.

Recently Montgomery et al (eLife. 11:e79258 (2022). doi: 10.7554/eLife.79258) reported that paternal chromosomes are repressed by genomic imprinting, resulting in functional haploidy even in diploid sporophytes. They illustrate how gametophyte-dominant species can regulate gene dosage through paternal chromosome inactivation and initiate the exploration of the link between life cycle history and gene dosage in Marchantia. The authors must refer to the paper above and discuss the difference/similarity of the Ectocarpos system in the manuscript. Is there any possibility that Ectocarpos has a similar silencing mechanism?

© 2024. Published by The Company of Biologists under the terms of the Creative Commons Attribution License (https://creativecommons.org/licenses/by/4.0/).

Page 6, the second paragraph: It is well known that dosage compensation is crucial to equalize the gene expression of the genes on the X chromosome in organisms with XX/XY.
In organisms of U/V, only UV is the chromosome combination seen in sporophytes. Gene dosage compensation is not generally required. If it is dosage compensation between gametophyte and sporophyte, it would be the problem mainly for autosomes and PAR. The reason for analyzing dosage compensation for SDR should be explained carefully.

In Figure 4, H3K79me2 was analyzed across the life cycle. The analysis of the chromatin status of PAR (and autosomes) in the sporophyte generation is technically impossible if there are no SNPs in PAR for U and V and (for maternal and paternal autosomes). Do authors assume that autosomes and PAR from male gametophyte and the female gamete has the same epigenetic modifications? If so, is the assumption justified by the previous observations?

The information on HMG-sex is limited. The reference (Luthringer et al. submitted) should be published before acceptance.

Minor point
Figure 1, D. Delete the hyphen from the label of the second panel.

First revision

Author response to reviewers' comments

Reviewer 1 Comments for the author
Comments were uploaded and are available as a PDF at https://submit-dev.biologists.org.

***** Reviewer 2 Advance summary and potential significance to field
Vigneau and co-authors reported the genetic interactions between the U and V chromosomes by analyzing transcription profile and chromatin status across the life cycle of the unique model brown algae Ectocarpus. They also analyzed those of the oro mutant that decouple developmental programs from ploidy. They revealed developmental regulation of the genes encoded in the sex-determining loci of the sex chromosomes. They provided convincing epigenetic data to compare the chromatin status of sexual phenotype of each generation of the life cycle. Overall, the study provided insights for understanding the genetic regulation of the UV sex chromosomes. Several suggestions are listed below for possible improvement of the manuscript.

Reviewer 2 Comments for the author
Sex chromosome dominance is a fundamental question in biology. However, the analysis of the oro:oro mutant is suggestive but not sufficient to conclude the sex chromosome dominance of the UV system in Ectocarpos. ORO is primarily required for proper sporophyte development (substrate adherence structure) since diploid oro;oro mutants are more closely related to haploid gametophytes. Although male sex determination was observed in oro;oro mutants, it is hard to conclude if the observed male phenotype is essentially due to the dominant character of V over U by oro mutation phenotype or by just independent from oro. What is the default sex of Ectocarpos? The observed negative correlation in transcript accumulation between ORO-SAM and HMG-sex might provide insight, but it is not strong evidence for the conclusion. Ideally, sex chromosome dominance in a UV system would be analyzed in aneuploid isolates with the U and V chromosomes in the gametophyte generation. Therefore, sex chromosome dominance should be still discussed carefully.

Re: We thank the reviewer for raising this important point. We had previously shown that V is dominant over U by using genetic approaches (Ahmed et al, Curr Biol, 2014) and our more recent work (Luthringer et al, Science, in press) validates this idea, and further demonstrates that HMG-sex is the male master sex determinant in Ectocarpus. The presence of this gene in UV diploid gametophytes (oro;oro) leads to activation of the male program, despite the presence of a U chromosome (Ahmed et al, Curr Biol, 2014). hmg-sex knockout in the oro;oro mutant (UV)

© 2024. Published by The Company of Biologists under the terms of the Creative Commons Attribution License (https://creativecommons.org/licenses/by/4.0/).

background, results in a reversal of the male sex phenotype to a female, sreongly arguing for a role of HMG-sex in being the master transcription factor that induces male sex. We thus believe that female sex is the 'background' sex in Ectocarpus. We have added this new information to the manuscript and, as suggested, we have carefully reworded suggestions of sex chromosome dominance in the revised version.

The paper demonstrating that HMG is the master sex determining gene is in press and we have now added the reference to the manuscript, which should make the narrative easier to follow.

Related to the comments above, The paper's concise title "Sex chromosome dominance in a UV sexual system," does not adequately reflect the results shown in the manuscript. Another reason that the title should be more informative is that this is an original research article, not a review article.

Re: We agree in hindsight the lack of detail in the old title. As suggested, we now propose a new title in the revised version that we hope is more reflective of our results.

The abstract should also be improved to describe the background information accurately. The first three sentences broadly state the importance of the subject of the study; the first and third sentences are correct, but the second sentence that starts with "In such organisms" is incorrect. Vascular plants have an alternation of generations, but most of them do not have sex chromosomes, and when they do, sex chromosomes are XX/XY (diploid system), not U and V. The sex-determination system by UV sex chromosomes needs to be accurately described so that the general readers should easily understand the differences from well-known XX/XY (ZW/ZZ) without confusion.

Re: We have added more context to the beginning of the abstract as rightly suggested by the reviewer. Note that following the guidelines of Development we had to reduce the number of words of the abstract to 180, therefore limiting the amount of information we can include.

Recently Montgomery et al (eLife. 11:e79258 (2022). doi: 10.7554/eLife.79258) reported that paternal chromosomes are repressed by genomic imprinting, resulting in functional haploidy even in diploid sporophytes. They illustrate how gametophyte-dominant species can regulate gene dosage through paternal chromosome inactivation and initiate the exploration of the link between life cycle history and gene dosage in Marchantia. The authors must refer to the paper above and discuss the difference/similarity of the Ectocarpos system in the manuscript. Is there any possibility that Ectocarpos has a similar silencing mechanism?

Re: We thank the reviewer for pointing out this oversight. We are aware of this work and it should have rightly been cited. We have now discussed this work further in the discussion. It is important to note that the life history and alternation of generations in Marchantia and Ectocarpus are very different. In Marchantia, the gametophyte is the dominant phase while the sporophyte phase is greatly reduced and viviparous (ie. dependent on the female gametophyte), initiating meiosis after just five cell divisions. In Ectocarpus, no vivipary exists since fertilisation is external and both stages are free-living, only slightly heteromorphic and live equally as long as each other.

Page 6, the second paragraph: It is well known that dosage compensation is crucial to equalize the gene expression of the genes on the X chromosome in organisms with XX/XY.
In organisms of U/V, only UV is the chromosome combination seen in sporophytes. Gene dosage compensation is not generally required. If it is dosage compensation between gametophyte and sporophyte, it would be the problem mainly for autosomes and PAR. The reason for analyzing dosage compensation for SDR should be explained carefully.

Re: We thank the reviewer for this comment. We realised we should have clarified more carefully what we mean with "dosage compensation". In animals with XX/XY or XO systems, X-linked genes need to be balanced in relation to autosomes in males and females because of the presence of 2 X copies in the female and one X copy in the male. Many XX/XY organisms (like mammals) inactivate one of the X in females, while XO organisms (like flies) double the transcription of the X in males. In the case of UV organisms, no chromosome scale dosage compensation occurs because males and females have either a V or a U . But at the level of single genes, some that are unique to either the

© 2024. Published by The Company of Biologists under the terms of the Creative Commons Attribution License (https://creativecommons.org/licenses/by/4.0/).

U or the V can only contribute half the dosage if required in a diploid context. So theoretically dosage compensation could take place at the level of such genes by doubling their expression to match ploidy of autosomal genes in the sporophyte. Our work does indeed show that at least one gene doubles its expression in the diploid stage and might thus be subjected to dosage compensation at the single gene level. The revised version of the manuscript now hopefully clarifies these aspects by specifying what we mean with dosage compensation.

In Figure 4, H3K79me2 was analyzed across the life cycle. The analysis of the chromatin status of PAR (and autosomes) in the sporophyte generation is technically impossible if there are no SNPs in PAR for U and V and (for maternal and paternal autosomes). Do authors assume that autosomes and PAR from male gametophyte and the female gamete has the same epigenetic modifications? If so, is the assumption justified by the previous observations?

Re: We thank the reviewer for raising these important questions. We cannot rule out that H3K79me2 profiles (and transcription more generally) might be biased between maternal and paternal autosomes and the PAR regions in the sporophyte stage of Ectocarpus, as has been shown in Marchantia. Again, given the stark differences in their life history (see above), it would be surprising if this were the case. Unfortunately, the strains used for the crosses to obtain the sporophyte show little genetic differences (Gueno et al. 2022), so we do not have sufficient genetic variation between the male and female gametophyte siblings to ask this type of question. We have added a note entertaining this possibility in the section dealing with paternal chromosome dosage in Marchantia.

Nevertheless, we believe that our analysis of H3K79me2 over the SDR regions is justified since these regions are specific to either the U or the V. The overall reduction of H3K79me2 along the SDRs is thus not potentially conflated by an extra copy in the genome, as it would along the autosomes. We have now toned down the idea of “global reconfiguration” and also mention the fact that we are unable to assess parent-of-origin effects in the sporophyte.

The information on HMG-sex is limited. The reference (Luthringer et al. submitted) should be published before acceptance.

Re: Our publication has now been accepted and a DOI is available, which we include in the revised manuscript.

<u>Second decision letter</u>

MS ID#: DEVELOP/2024/202677

MS TITLE: Interactions between U and V sex chromosomes during the life cycle of *Ectocarpus*

AUTHORS: Jeromine Vigneau, Claudia Martinho, Olivier Godfroy, Min Zheng, Fabia Haas, Michael Borg, and Susana M. Coelho

ARTICLE TYPE: Research Article

I am happy to tell you that your manuscript has been accepted for publication in Development, pending our standard ethics checks.

© 2024. Published by The Company of Biologists under the terms of the Creative Commons Attribution License (https://creativecommons.org/licenses/by/4.0/).
